# The effect of mobile phone addiction on perceived stress and mediating role of ruminations: Evidence from Chinese and Czech university students

**DOI:** 10.3389/fpsyg.2022.1057544

**Published:** 2022-12-19

**Authors:** Hongyang Liu, Jan Sebastian Novotný, Lucie Váchová

**Affiliations:** ^1^Department of Psychology and Abnormal Psychology, Faculty of Education, Palacký University Olomouc, Olomouc, Czechia; ^2^Translational Neuroscience and Aging Program, Center for Translational Medicine, International Clinical Research Center, St. Anne’s University Hospital, Brno, Czechia

**Keywords:** mobile phone addiction, rumination, perceived stress, university students, mediation, cross-cultural differences, Czech Republic, China

## Abstract

**Introduction:**

The rise in the capabilities of mobile devices and the associated increase in the proportion of time we spend on them has not only positive benefits but also several risks, including mobile phone addiction and its consequences. The complex mechanisms of the impact of this addiction on mental health, especially in a cross-cultural context, however, remain relatively unknown. The aim of this cross-cultural study was to investigate the mediating role of rumination on the association between mobile phone addiction and perceived stress.

**Methods:**

A population of 358 Chinese and 282 Czech university students was tested using a battery of validated psychological tests that included a short version of the Smartphone Addiction Scale, the Ruminative Response Scale, and the Perceived Stress Scale.

**Results:**

The results showed significant cross-cultural differences with Czech students manifesting greater rumination (*d* = 0.79) and perceived stress (*d* = 0.42) and Chinese students showing greater mobile phone addiction (*d* = 1.01). Mediation analyses showed that the effect of mobile phone addiction on stress levels was mediated through the rumination in both populations (45.6% and 80.9% of the explained variance for Chinese and Czech students, respectively) and did not differ between the two countries (estimate of difference [95%CI] = −0.052[−0.166, 0.037], *p* = 0.27). In contrast, the significant direct effect of mobile phone addiction on perceived stress was only present in Chinese students, where it was marginally larger than the indirect effect. In Czech students, the direct effect was not manifested and the difference between countries was significant (estimate of difference [95%CI] = 0.242 [0.035, 0.413], *p* < 0.001). In all of the cases, the association between the variables was positive, i.e., as one grew, so did the other. Finally, a moderated-mediation analysis confirmed that country of origin significantly moderated only the direct relationship between mobile phone dependence and perceived stress (*p* = 0.002).

**Discussion:**

These results suggest that the mechanism of interaction between excessive mobile phone use and perceived stress is culturally conditioned, which may limit the transferability of research findings in a global context and requires further cross-cultural studies.

## Introduction

The evolution of mobile device capabilities has been almost ‘meteoric’ in recent decades, and today’s mobile phones are capable of doing things that were reserved only for computers a few years ago. This makes many people use their phones more and more as their primary technological tool for communication, gaming, reading, studying or shopping. Along with this, we can see a significant increase in the role of mobile phones in social networking. According to App Annie (2020), a mobile data and analytics company, which recently released the State of Mobile 2022 report, global mobile device usage in 2021 is at a record high of 3.8 trillion hours (Olson et al., 2022). Furthermore, a meta-analysis by McGill University showed that Chinese users ranked first in the world in mobile phone addiction rates compared with European countries such as Germany and France (Olson et al., 2022), as China’s young population uses mobile phones too often and for too long (Liu et al., 2017). These so-called ‘heads down’ groups of university students can be seen everywhere.

Although mobile phones offer a number of benefits and conveniences for university students (and the general population), such as the ability to communicate quickly with family, teachers and classmates (Park and Lee, 2012), the possibility of meeting, interacting and communicating with distant friends (Auter, 2007), a tool for studying and filling leisure time, a means of reducing stress and tension as well as the ability to purchase goods that are not available locally (Hossain, 2019), the excessive use of the phone can also have a number of negative impacts. Inappropriate use of mobile phones may hinder face-to-face communication (Krasnova et al., 2016) and reduce interpersonal intimacy (Roberts and David, 2016). Using mobile phones in class and studying can be a distraction for students and be a cause of academic procrastination or lower academic performance (Balkis, 2013; Byun et al., 2013; Ibrahim et al., 2018). Moreover, excessive mobile phone use can result in physical and psychological problems or the development of a mobile phone addiction.

Mobile phone addiction was first described by Young as a type of technology addiction, which further includes for example Facebook addiction, internet addiction or video games addiction (Young, 2007). It is considered as an addictive behavior, as it encompasses some of the general characteristics of addictive behavior: lack of self-control, withdrawal symptoms, and tolerance (Griffiths, 1995; Griffiths, 2005). Mobile phone addiction can have a variety of negative consequences. Excessive smartphone use has been linked to a higher risk of anxiety and depression (Thomée et al., 2011; Rosen et al., 2013; Firth et al., 2017; Matar Boumosleh and Jaalouk, 2017; Lei et al., 2020; Cheng et al., 2021; Ratan et al., 2021), poorer health (Schoeni et al., 2015), sleep problems, pain and migraine, cognitive impairment, poorer self-esteem (Wacks and Weinstein, 2021), fatigue (Feng and Sun, 2022), neurological disorders (Ratan et al., 2021) or increased perceived stress (Chiu, 2014; Kuang-Tsan and Fu-Yuan, 2017; Reinecke et al., 2017; Gao et al., 2018; Xu et al., 2019), particularly in adolescents and young adults (Matar Boumosleh and Jaalouk, 2017; Cha and Seo, 2018; Loleska and Pop-Jordanova, 2021). In fact, the significant increase of this type of addiction in the young population is emerging as one of the priority public health issues. Moreover, despite a number of studies examining this issue, comparative studies accounting for the effects of different cultural backgrounds are still lacking.

Although the link between mobile phone addiction and stress is well documented, the mechanisms underlying this relationship are not yet fully understood. Given that increased stress in the context of this addiction occurs in conjunction with a lack of coping mechanisms, depression and increased susceptibility to addiction (Matar Boumosleh and Jaalouk, 2017), ruminative thinking may be one of the mechanisms explaining the above relation. Ruminative thinking (or rumination) is the tendency to focus attention on the symptoms of one’s distress and on its possible causes and consequences as opposed to its solutions. As such, it is perceived as a dysfunctional cognitive coping strategy (Nolen-Hoeksema et al., 2008; Samtani and Moulds, 2017) that represents an excessive and intrusive fixation on negative experiences and feelings. Several studies have suggested that rumination is a consequence of internet addiction (Davis, 2001) as individuals with higher addiction have a deficit in the ability to regulate affective emotions, leading to a stage of maintained negative affectivity with lower positive affectivity (Joormann and Quinn, 2014). The indirect effect of mobile phone addiction also plays a role as increased smartphone time negatively affects a range of offline activities, including interpersonal relationships and academic achievements, which increases the stress load (Kuss and Griffiths, 2011) which in turn can further saturate negative affectivity and rumination (Liu et al., 2017). Furthermore, rumination has also previously been linked to stress levels (Kaiseler et al., 2017; Lian et al., 2021) within the diathesis stress model in which rumination caused by a negative life context (e.g., addiction) may increase subsequent experiences of stress and other psychopathologies such as depressive symptoms (Morrison and O’Connor, 2005).

In addition, the issue of mobile phone addiction and its negative consequences has become even more important in the last 2 years as the COVID-19 pandemic has had a number of negative impacts on humans. The reduction in the possibilities for face-to-face interaction, lockdowns and social distancing (Nurunnabi et al., 2021; Viner et al., 2021), increased uncertainty about the future, financial implications (Cao et al., 2020; Russo and Terraneo, 2020), health and mental health risks (Son et al., 2020; Browning et al., 2021; Fruehwirth et al., 2021; Křeménková et al., 2021; Xu et al., 2021) as well as the inevitability of having to spend a great amount of time online which is also flooded with more negative content (Ratan et al., 2021; Serra et al., 2021) may play a role in the increased development of mobile phone addiction and the magnification of its negative consequences and mental health issues in general, especially in individuals who have less resources to cope with challenging situations and adversities.

Surprisingly, although previous studies have both described the mediating role of rumination in the context of mobile phone addiction (Wang et al., 2018; Lian et al., 2021; Liu et al., 2021; Li et al., 2022; Peng et al., 2022) and have indicated cross-cultural differences in perceived stress (Lee et al., 2022; Mohamed et al., 2022), ruminative thinking (Murdock et al., 2019) and mobile phone addiction (Yang et al., 2019; Olson et al., 2022), there is no direct evidence available on the cultural conditioning of this mediating relationship. To explore the above-mentioned mechanism and in part fill the gap in understanding the role of cross-cultural differences, the aim of this study was to examine the mediating role of ruminative thinking on the relationship between mobile phone addiction and perceived stress in two different cultural settings and to compare them against each other.

## Materials and methods

### Study design and sample

At the beginning of this cross-cultural study, we approached 756 students from the Faculty of Education, Palacký University *via* their official university emails and 413 students from the Faculty of Educational Sciences, Chongqing Normal University in Sichuan province *via* Wechat social app concerning their participation in the study. Of these, 282 (68.3%) Czech and 358 (47.4%) Chinese students were eventually involved. Data were collected using an online questionnaire (Google Forms in Czech Republic and Wenjuanxing in China) in two identical forms in Czech and Chinese. Data collection took place from February to April 2022.

### Measure and instruments

Mobile phone addiction (‘mpa’) was measured using the Short Version of the Smartphone Addiction Scale (SAS-SV, Kwon et al., 2013). The scale consists of 10 items rated on a 6-point Likert scale (1 = totally disagree, 6 = totally agree), with higher scores indicating a higher level of mobile phone addiction. McDonald’s omega in this study was good to excellent at 0.93 and 0.86 for the Czech and Chinese samples, respectively.

Perceived stress (‘pss’) was measured using the Perceived Stress Scale (PSS, Cohen et al., 1983; Cohen and Williamson, 1988). The scale has 10 items and is scored on a 5-point Likert scale ranging from 1 = never to 5 = always. A higher the score indicates increased perceived stress. Studies have shown good reliability in different cultural settings. McDonald’s omega in this study was good at 0.78 and 0.77 for the Czech and Chinese samples, respectively.

Rumination (‘rmn’) was assessed using the Rumination Response Scale (RPS, Nolen-Hoeksema, 1991). This 22-item scale measures the tendency to ruminative thinking. The items are answered on a 4-point Likert scale ranging from 1 = never to 4 = always, with a higher total score reflecting a greater tendency toward ruminative thinking. McDonald’s omega in this study was excellent at 0.94 and 0.96 for the Czech and Chinese samples, respectively and the tool demonstrated good applicability in the Chinese population (Li et al., 2019).

The English versions of SAS-SV and RPS were initially translated to Czech by the standard double-blind approach (two independent translations to Czech, two back-translations to English by a native-speaker and a psychologist, comparison of the original and back-translated versions). The Czech version of the PSS and Chinese versions of all questionnaires were available from previous studies.

### Data analysis

No missing values were detected. The reliability of each instrument (separately for each country) was verified using McDonald’s omega. Due to the non-normal data distribution, all variables were standardized to Z-scores prior to the analyses. Associations between variables were first analyzed using Pearson’s correlation, differences between correlation coefficients by z-test with Fisher’s r-to-z transformation, and group differences using Welch *t*-test. Complex interactions between variables were analyzed using a series of casual mediation analyses (with 1,000 simulations) and the effect of country of origin on the mediation effect was then tested using a test of significant differences in direct and indirect effects (with 200 simulations) and a moderated-mediation analysis (Tingley et al., 2014). Data analyses and visualizations were performed using RStudio (v. 1.4.1717 with R environment v. 4.1.3) using cocor, dplyr, flexplot, GGally, ggplot2, jmv, JSmediation, mediation, rstatix, and stats packages.

### Ethical consideration

The authors assert that all of the procedures contributing to this work comply with the ethical standards of the relevant national and institutional committees on human experimentation and with the Helsinki Declaration of 1975, as revised in 2008. All of the participants were informed of the confidentiality of their answers and signed an online informed consent form prior to the completion of the questionnaire. No specific information enabling the identification of specific students (such as IP address, student name or ID number, specific field of study, etc.) was obtained as part of the online data collection. The research protocol of the study was approved by the Ethics Committee of the Faculty of Education.

## Results

### Sample demographics

The research population consisted of two cohorts of university students. The Czech population consisted of 282 students (mean age ± SD, 25.1 ± 7.5, 87.6% females), while the Chinese population consisted of 358 students (mean age ± SD, 21.6 ± 2.0, 68.2% females). The baseline characteristics of both samples are shown in Table 1.

**Table 1:** Main characteristics of the research cohorts.

	China	Czech Republic
*N*	358	282
Mean age ± SD	21.6 ± 2.0	25.1 ± 7.5
Sex, *n* [%]		
Male	114 [31.8%]	35 [12.4%]
Female	244 [68.2%]	247 [87.6%]
Level of study, *n* [%]		
Bachelor’s	135 [37.7%]	203 [72%]
Master’s	223 [62.3%]	79 [28%]
Residence, *n* [%]		
Home	231 [64.5%]	171 [60.6%]
Dormitory	127 [35.5%]	111 [39.4%]

### Associations between variables

**Figure 1:**
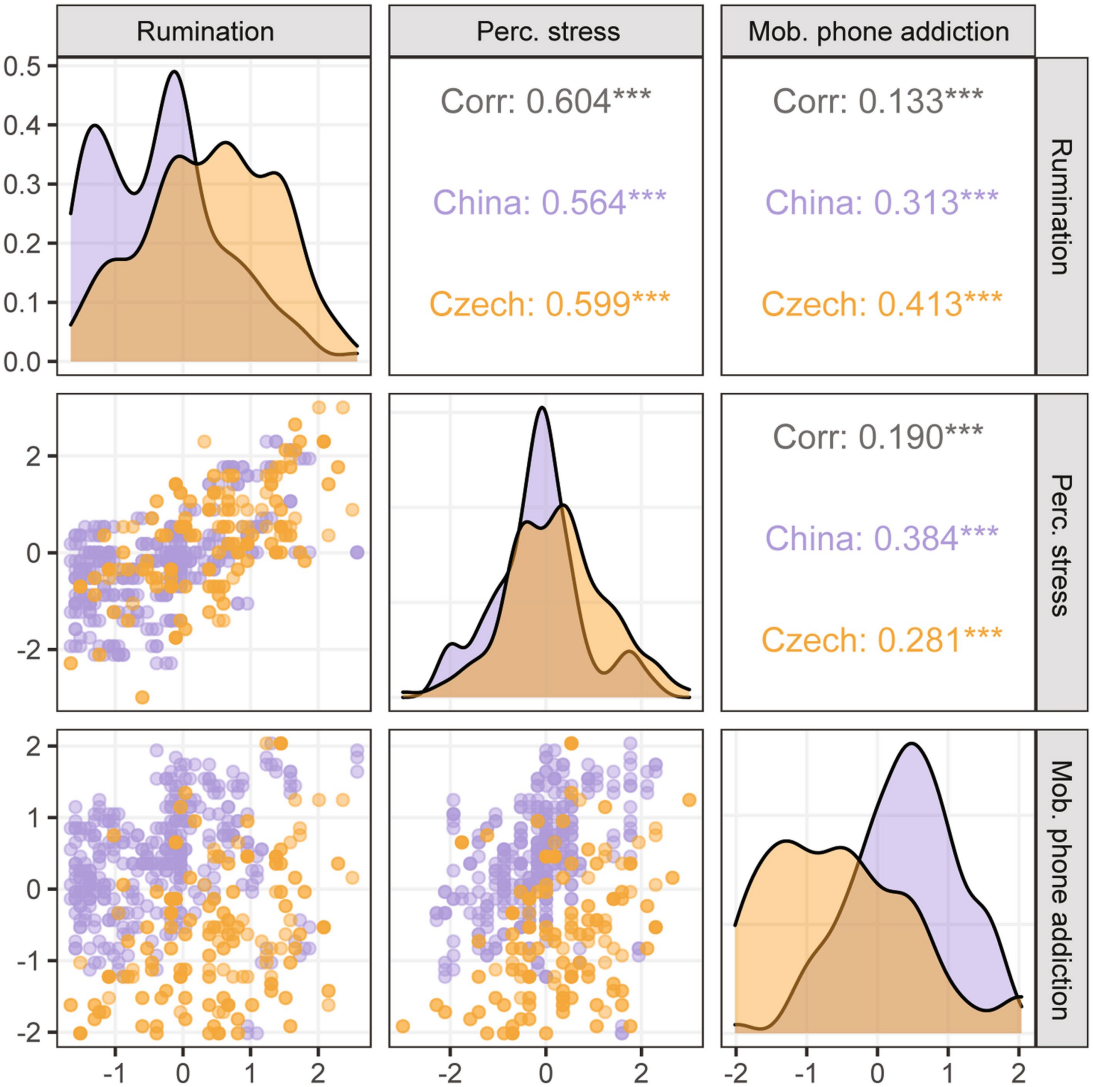
Correlations between main outcomes. Correlation matrix showing the density plot of the z-scores of each variable, the scatterplot of the distribution of values in both countries and the strength of the correlations between the variables. ***p < 0.001.

The initial analysis showed significant correlations between all three variables across the dataset and within both countries (Figure 1) with small to medium effect sizes for the relationship between mobile phone addiction and rumination and perceived stress and large effect sizes for the relationship between rumination and perceived stress. However, the correlation coefficients did not differ between the two countries (*P*_rmn ~ pss_ = 0.146, *P*_mpa ~ rmn_ = 0.152, *P*_rmn ~ pss_ = 0.502).

Furthermore, we also observed significant cross-cultural differences in rumination [*t*(590.43) = −9.930, *p* < 0.001, *d* = 0.79 (moderate)] and perceived stress levels [*t*(568.18) = −5.270, *p* < 0.001, *d* = 0.42 (small)] with higher values in Czech students and in mobile phone addiction [*t*(497.67) = 12.483, *p* < 0.001, *d* = 1.01 (large)] with higher levels in Chinese students.

### Mediation analysis

**Figure 2:**
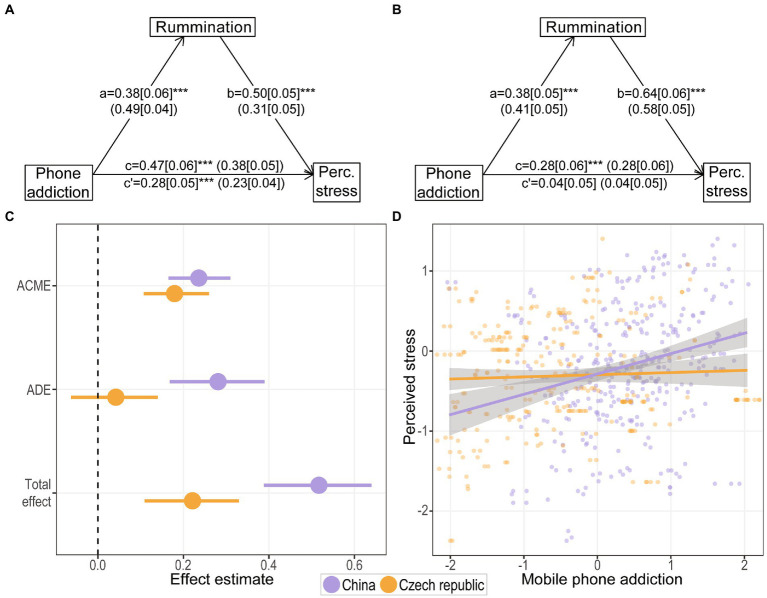
Casual mediation and moderated–mediation models. Simple mediation diagrams showing a, b, c, and c’ path coefficients for **(A)** Chinese and **(B)** Czech students, representing unstandardized regression weights with standardized regression weights in parentheses. The square brackets contain the SE. ***p < 0.001. The c path coefficient represents the total effect, the c-prime path coefficient refers to the direct effect. **(C)** Country–specific estimates of direct and indirect effects of mobile phone addiction on perceived stress with rumination as the mediator. **(D)** Direct effects of mobile phone addiction on perceived stress in both countries considering the mediating effect of rumination.

Subsequently, we examined the effect of mobile phone addiction on the level of perceived stress and the possible role of rumination as a mediator in this relationship. A casual mediation analysis showed a significant both direct and indirect effect in Chinese students (Table 2; Figures 2A,C). The direct effect of mobile phone addiction on perceived stress was marginally greater with higher perceived stress and greater rumination associated with greater phone addiction. The mediation effect explained 45.6% of the net total effect. In contrast, Czech students did not show a direct effect of phone addiction on perceived stress. Here, mobile phone addiction was positively related only to rumination, through which it also influenced the level of perceived stress (Table 2; Figures 2B,C). This mediation effect explained 80.9% of the net total effect.

**Table 2:** Estimates of direct and indirect effects with quasi–Bayesian confidence intervals for both countries.

	China		Czech Republic	
	Estimate [95%CI]	*p*	Estimate [95%CI]	*p*
ACME	0.236 [0.17, 0.31]	<0.001	0.179 [0.11, 0.26]	<0.001
ADE	0.281 [0.17, 0.39]	<0.001	0.042 [−0.06, 0.14]	0.41
Total effect	0.517 [0.39, 0.64]	<0.001	0.221 [0.11, 0.33]	<0.001
Proportion mediated	0.456 [0.33, 0.61]	<0.001	0.809 [0.50, 1.50]	<0.001

ACME – Average Causal Mediated Effect, ADE – Average Direct Effect.

### Cross-cultural differences

To verify the effect of students’ country of origin on the observed differences in mediation effects, we statistically tested these differences. The results showed that the indirect effects did not differ between the two groups of students (estimate of difference [95% CI] = −0.052 [−0.166, 0.037], *p* = 0.27), while the direct effect was significantly larger for Chinese students (estimate of difference [95% CI] = 0.242 [0.035, 0.413], *p* < 0.001).

This was further confirmed by the moderated-mediation analysis (with students’ country of origin as the moderator), which showed that country of origin did not moderate the relationship between phone addiction (independent variable) and rumination (mediator; a*Mod: t(636) = 0. 03, *p* = 0.976) or the relationship between rumination and perceived stress (dependent variable; b*Mod: *t*(634) = 1.87, *p* = 0.062) but significantly moderated the direct relationship between mobile phone addiction and perceived stress (*c*’*Mod: *t*(634) = 3.18, *p* = 0.002; Figure 2B).

## Discussion

The aim of this cross-cultural study was to investigate the relationship between mobile phone addiction, ruminative thinking, and perceived stress and the mediating role of rumination in two different cultural settings. The rationale for this study arose from three observations. First, previous studies have provided evidence for the mediating role of ruminative thinking in the context of mobile phone addiction and mental health (Wang et al., 2018; Lian et al., 2021; Liu et al., 2021; Li et al., 2022; Peng et al., 2022). Second, there is separate evidence for cross-cultural differences in perceived stress (Lee et al., 2022; Mohamed et al., 2022), ruminative thinking (Murdock et al., 2019) and mobile phone addiction (Yang et al., 2019; Olson et al., 2022). And third, and most importantly, there is surprisingly no direct evidence of cultural conditioning of the aforementioned mediating relationship. This study thus seeks to fill this gap in our knowledge.

We observed a significant small to moderate positive relationship between these variables, which was the same for both student populations. These findings are consistent with previous studies that described an adverse effect of mobile phone addiction on perceived stress (Chiu, 2014; Kuang-Tsan and Fu-Yuan, 2017; Reinecke et al., 2017; Gao et al., 2018; Xu et al., 2019) and an increase in rumination (Morrison and O’Connor, 2005; Joormann and Quinn, 2014; Kaiseler et al., 2017; Liu et al., 2017; Lian et al., 2021). We hypothesize that this effect is exerted directly through the potential influence of ‘withdrawal’ symptoms on internal emotional turmoil as well as indirectly through the negative impact of excessive smartphone time on other activities in students’ lives, including their studies. Other potential influence may be that for some individuals their mobile device is an important surrogate tool for their social functioning or for maintaining self-concept and self-esteem. However, Li, Liu, and Dong showed that problematic smartphone use was rather associated with lower self-esteem, depression and interpersonal confidence (Li et al., 2019). In line with Billieux et al. (2015) it appears that although students in some cases resort to excessive online communication, messaging and social media as a means of fulfilling their needs and gaining reassurance and self-worth, individuals predisposed to depressive and anxiety symptoms in particular are in fact more likely to promote a vicious cycle of constantly seeking new and novel sources of self-affirmation and acceptance (Elhai et al., 2019). This can be particularly problematic when the content consumed on a mobile phone is dominated by negative feedback and information, which the individual finds harder to process due to poorer coping skills (Matar Boumosleh and Jaalouk, 2017). It should also be taken into account that the ongoing Covid-19 pandemic may have affected the identified relationships. This potential effect will be summarized below in the mediation analysis section.

We also observed differences between the two student populations. Consistent with recent studies, Chinese students showed significantly higher cell phone addiction (App Annie, 2020; Olson et al., 2022). One reason for this difference may be the different cultural and philosophical backgrounds in the two countries. While the Czech Republic is considered a ‘Western’ country with many features of a consumerist society, due to its recent past (satellite country of the USSR and the restrictive communist regime), a certain delay in the development of consumerism and due to the effect of a relatively small land area and still sustaining face-to-face interpersonal contacts and bonds, the intensity and depth of connection to the ‘online world’ of mobile phones and social networks is still rather weaker. China, on the other hand, as a typical Eastern country with a long-term farming civilization, is characterized by significantly higher collectivism compared to the Western world (Triandis and Gelfand, 2012). Several studies have found that individuals with a higher tendency to collectivism have higher interpersonal sensitivity, that is, the tendency to be overly sensitive to the emotional and behavioral performance of others, especially the criticism and rejection of others (Yamaguchi, 1994; Scharf et al., 2017) and may experience emotional distress such as social anxiety and reluctance to express their emotions and social needs. The following three characteristics of the mobile phone network can make these interpersonally oversensitive individuals more likely to become dependent: anonymity, convenience and escape (Xia et al., 2003). Various social apps have made mobile social networking more convenient because the anonymity of mobile social networking allows people to do what they want without worrying too much about what they say or do will bring harm to them; the convenience gives people more opportunities to connect with strangers and have supportive social relationships. This is more important for people who cannot meet their social needs in reality, so people who are interpersonally sensitive are more likely to escape reality and immerse themselves in mobile social interaction (You et al., 2019). Furthermore, influenced by collectivism and Confucianism, the Chinese attribute great importance to the synchronicity of the collective and the opinion of their peers (Yum, 1988). There is a phenomenon among young Chinese people nowadays that they gather together among their classmates or friends to play online games on mobile phones and those who are reluctant are forced to participate by the peer pressure (Han and Qi, 2005). The need not to disappoint their peers drives these young people to participate in these activities but as the frequency of mobile gaming increases, so does their level of stress (Assor et al., 2020). Besides higher collectivism, other possible contributing factors may be the significantly larger size of China and more frequent long distances between students and their family and friends, which can be overcome regularly only by using online tools. Chinese students may therefore be more likely to use mobile phones to kill leisure time, relieve stress and as entertainment means. Also, the effect of the Covid-19 pandemic circumstances, which will be discussed below, may have played a role here.

Mediation and moderated-mediation analyses revealed cross-cultural differences between the two groups of student populations. While the effect of mobile phone addiction on stress in Chinese students is approximately split in half between a direct effect and a mediating effect through the level of rumination, in Czech students the stress levels are only affected through ruminative thinking (explaining a full 80% of the variance in perceived stress). For this group of students, the problematic smartphone use is not directly related to the level of stress.

The observed mediating effect of rumination is consistent with similar studies which also observed the mediating role of rumination on the relationship between mobile phone addiction and stress (Liu et al., 2017; Lian et al., 2021) and in the opposite direction and under similar circumstances of the Covid-19 pandemic (Peng et al., 2022). These results suggest that maladaptive behaviors associated with excessive smartphone use negatively affect students’ emotional state and their response to adverse circumstances in the form of increased rumination (Nolen-Hoeksema, 1991). This negative attunement then contributes to a more pronounced experience of stress. An example of such a sequence is the effect on self-image in individuals with lower self-esteem and a stronger external source of self-concept (which is more typically the case for adolescents and young adults). A greater need for external validation of self-worth through acceptance on social media increases the need for activity and consumption of content on these apps. Negative feedback or the mere absence of sufficient feedback can increase a person’s risk of developing ruminative thoughts, which in turn increases the drive to achieve their goal through greater activity on social media, thereby increasing their stress levels or rates of depression (Feinstein et al., 2013). Another source of more negative emotionality and rumination and increased stress as a result of excessive smartphone use may be the so-called fear of missing out (FOMO) phenomenon which is based on the conviction that one is either not in the know or missing out on information, events, experiences or life decisions that could make his/her life better and which has been repeatedly identified as an element of mobile phone addiction and a possible source of rumination and stress in both European and Chinese students (Yang et al., 2019, 2021; Li et al., 2020; Brailovskaia et al., 2021).

The direct effect of mobile phone addiction on perceived stress was observed only in Chinese students. This difference may have several causes. As we observed, the degree of mobile phone addiction was lower among Czech students. As a result, the negative impact of the symptoms of this addiction, such as worrying, withdrawal symptoms or escape to the online environment with a reduction of activities in the offline world, could be lower, which then does not create as many daily-life disturbances (Pavia et al., 2016). The richer variety of direct interpersonal activities available to Czech students compared with Chinese students, for whom these opportunities seem to be reduced, may also have played a role. Finally, social peer pressure may also play a role in this context, as has already been suggested above in the context of the phenomenon among young Chinese of coming together to play online games. The perceived need to engage in these activities (also in the context of the collectivism principle mentioned above), even when one is not realistically interested, leads to a person forcing oneself to engage (Han and Qi, 2005; Assor et al., 2020). However, the increasing frequency of these activities raises, among other things, the pressure on the person and his or her perceived stress. Regardless, these results suggest that the socio-cultural background needs to be taken into account in research and prevention and intervention of problematic smartphone use and mobile phone addiction.

Finally, the potential impact of the Covid-19 pandemic circumstances should also be considered. Previous studies have shown that the pandemic had a negative impact on the mental well-being of many people, whether in terms of increased stress, anxiety or depression, which is closely associated with ruminative thinking (Daly et al., 2020; Novotný et al., 2020; Ye et al., 2020; Ramiz et al., 2021; Ettman et al., 2022; Jia et al., 2022; O’Connor et al., 2022). Similarly, the pandemic led to the necessity to spend much more time in the online environment with the online space being heavily laced with negative content. On the other hand, the data collection took place after 2 years of the pandemic, during which students had to cope with a number of adversities such as disruptions in their studies and the transition to online learning, uncertainty about their future studies and careers as well as difficulties at the societal level such as curfews, high numbers of infections, hospitalizations and deaths, financial implications, etc. We can assume that students developed strategies to cope with these issues during the pandemic. Moreover, by the time of data collection, the restrictive measures in both countries were already relatively relaxed, lectures were carried out face-to-face and students could already meet and travel relatively freely (except to places with a more serious situation in China such as Shanghai). Based on this, we hypothesize that the Covid-19 pandemic did not directly affect the observed relationships between the variables but rather accentuated the inter-individual differences between students depending on the availability of coping resources (Al Qudah et al., 2021; Kayis et al., 2021; Kovács et al., 2021). This is supported by the fact that the relationships reported in the present study and the mediating effect of rumination have been documented in studies both before (Liu et al., 2017; Lian et al., 2021) and during the Covid-19 pandemic (Peng et al., 2022).

### Study limitations

The study has several limitations. First, women were over-represented in the research population, which may have had a partial effect on the relationships found. The main cause of this sex imbalance is the fact that faculties of education are mostly attended by women (regardless of country). Second, the cross-sectional nature of the study does not allow for a direct assessment of causality between the variables. Third, the research population consisted of students from faculties of education, which may have limited the ecological validity of the findings. Fourth, the use of the Wechat online tool in recruiting the respondents may have affected the representativeness of the sample (particularly in the context of the pattern and frequency of participants’ online presence). However, given that this tool is commonly and massively used by Chinese students, we do not think that potential respondents who are not online would be dropped by this approach. Finally, rumination and stress may be influenced by a number of other factors that were not included in this study and could potentially explain the mechanisms under investigation in greater depth. Future studies with more equal representation of both sexes, a more diverse sample, a wider range of variables and possibly of a longitudinal design should be carried out to further confirm our findings. The general and long-term validity of the results also needs to be verified in stable non-pandemic conditions.

### Conclusion

This cross-cultural study is one of the few to examine the interaction between mobile phone addiction, ruminative thinking and perceived stress in different student populations, providing important insights into the role of the socio-cultural background. We observed that Czech students showed higher levels of rumination and perceived stress, while Chinese students showed significantly higher mobile phone addiction. Furthermore, the results showed that the effect of higher mobile phone addiction on perceived stress is mediated through rumination in both populations, as mobile phone addicted students exhibited greater excessive fixation on negative feelings, which then resulted in increased perceived stress. However, only Chinese students also demonstrated a direct association between mobile phone addiction and perceived stress levels.

These results suggest that the mechanism of interaction between excessive mobile phone use, ruminative thinking and perceived stress is culturally conditioned, which may limit the transferability of research findings in a global context. Given that smartphone use rates have been significantly increased in recent years, may have been further greatly exacerbated by the Covid-19 pandemic and are becoming a priority public health issue, particularly in the young population, a better understanding of cross-cultural differences is crucial for effective prevention and intervention.

## Data availability statement

The raw data supporting the conclusions of this article will be made available by the authors, without undue reservation.

## Ethics statement

The studies involving human participants were reviewed and approved by Ethics Committee of the Faculty of Education, Palacký University Olomouc. The patients/participants provided their written informed consent to participate in this study.

## Author contributions

LV and HL collected data. JN made the statistical analysis. HL and JN wrote the draft of the manuscript. All authors conceived the idea of this study, contributed to the revision of the manuscript for important intellectual content, and reviewed the manuscript. All authors contributed to the article and approved the submitted version.

## Funding

The study was supported by the Palacký University in Olomouc, grant numbers IGA_PDF_2022_004 and IGA_PDF_2022_028.

## Conflict of interest

The authors declare that the research was conducted in the absence of any commercial or financial relationships that could be construed as a potential conflict of interest.

## Publisher’s note

All claims expressed in this article are solely those of the authors and do not necessarily represent those of their affiliated organizations, or those of the publisher, the editors and the reviewers. Any product that may be evaluated in this article, or claim that may be made by its manufacturer, is not guaranteed or endorsed by the publisher.
